# Making fetch happen: Prevalence and characteristics of fetching behavior in owned domestic cats (*Felis catus*) and dogs (*Canis familiaris*)

**DOI:** 10.1371/journal.pone.0309068

**Published:** 2024-09-04

**Authors:** Mikel M. Delgado, Judith L. Stella, Candace C. Croney, James A. Serpell

**Affiliations:** 1 Department of Comparative Pathobiology, College of Veterinary Medicine, Purdue University, West Lafayette, Indiana, United States of America; 2 Department of Clinical Sciences & Advanced Medicine, School of Veterinary Medicine, University of Pennsylvania, Philadelphia, Pennsylvania, United States of America; University of Lincoln - Brayford Campus: University of Lincoln, UNITED KINGDOM OF GREAT BRITAIN AND NORTHERN IRELAND

## Abstract

Both cats and dogs fetch, but the likely function(s) of this behavior for each species have not been compared. In this study, we assessed data from online surveys of cat and dog behavior (Fe-BARQ; C-BARQ) completed by cat (N = 8224) and dog owners (N = 73724). We assessed responses to the items "Plays ‘fetch’; likes to retrieve thrown objects or toys" (Fe-BARQ) and “Will ʻfetchʼ or attempt to fetch sticks, balls, or objects” (C-BARQ). Cats and dogs described as "sometimes," "usually" or "always" fetching were categorized as fetchers. Regression models were used to examine which animal-related (e.g., sex, age) and environmental factors best predicted fetching, and chi-square tests were used to explore the effect of breed on fetching behavior. Fetching was reported in 40.9% of cats and 77.8% of dogs. In cats, fetching was correlated with play and activity. In dogs, fetching was correlated with overall trainability. In both cats and dogs, being female, older, living with (other) dogs, and having health problems decreased the likelihood of fetching. Breed effects were observed in both species, with fetching more prominent in cat breeds originating in the Far East (e.g., Burmese, Siamese, and Tonkinese) and in dog breeds from the Retriever, UK Rural, Poodle, Pointer and Spaniel clades. We discuss the results in the context of domestication history of both cats and dogs and posit several hypotheses about why fetching behavior is observed in both.

## Introduction

Fetching is a behavior that is routinely observed in companion animals, most notably in dogs (*Canis familiaris*), which usually entails the retrieval of an object that has been thrown by a human. It is likely that dogs were selected for retrieving behavior during domestication, probably in the context of human hunting with projectile weapons [1]. Although it is assumed that many, if not most dogs are capable of fetching, some breeds, such as gun dogs, appear to have a stronger disposition for retrieving or “fetching” than others and these breed differences were likely selected for [2].

Fetching is one of the most frequently observed forms of play between dogs and humans [3]. Although often considered a play behavior, an owner may ask a dog to fetch a specific object, making the behavior a “task” based on the owner’s social cues and communication. It is assumed that some level of cooperation or “willingness to help” is expected from the dog in this context [4].

Although fetching behavior is observed in individual cats (*Felis catus*) [5], it is more difficult to make a case that the behavior is a product of functional selection for retrieving objects, as in dogs. Recent findings related to the social and play behaviors of cats indicate they have a considerable capacity for bonding with humans and responding to human social cues [e.g., 6, 7]. Play behavior has been observed in cats of varying ages, and object play, which has a relationship to predatory behavior, emerges as kittens are weaning at around four to five weeks of age [8]. Much like play in dogs, some play behaviors in cats may also have a human-directed social component [9].

All aspects of the fetching sequence likely relate to predatory behavior. Cats are stalk-and-rush hunters, engaging in short-distance chasing of moving prey [10]. Cats will carry prey away from the kill-site, perhaps to a safer location, or in the case of queens, carrying weakened prey to their offspring to promote the acquisition of hunting skills [e.g., 11]. Wolves (*Canis lupus*) and African wild dogs (*Lycaon pictus*) are known to engage in long-distance pursuit hunting of large ungulates [12] and wolves have been documented to engage in more stalk-and-rush type hunting of beavers [13]. Little is documented about the hunting strategies used by feral or free-roaming dog populations. Feral dogs have been reported to exhibit chase behavior and high levels of barking in loosely coordinated hunting groups that were ultimately unsuccessful [14]. They have also been documented to prey on rodents and rabbits but how they hunt them is not well understood [14]. Based on the existing literature, it is likely that both cats and dogs exhibit some behavioral plasticity in predatory behavior influenced by habitat and prey type.

Fetching behavior comprises a sequence of behaviors that reflect a level of synchrony between human and companion animal [15]; the human and pet must have each other’s attention and engage in turn-taking. The human must express intention to throw the toy when the pet is ready; the dog or cat must chase the thrown item, then they must pick up and carry the item in their mouth, returning the toy to the human to be thrown again. These behaviors may occur independently of one another, or in different combinations. For example, a cat could chase a thrown item and carry it to a location instead of returning it to the person who threw it. A dog could pick up and carry an item to a human, regardless of whether the item was thrown (e.g., a dog who has been trained to “fetch” their owner’s slippers).

A recent survey-based study of cat owners (n = 924) revealed details about the fetching behavior of 1154 cats. The survey specifically solicited owners of cats who displayed fetching behavior. Some characteristics of fetching behavior identified included a typical age of onset (less than 1 year of age, n = 701, 1–7 years of age n = 415) and frequency of fetching behavior (i.e., the number of fetching sessions within a month). The majority (94.4%) of cats who fetched offered the behavior spontaneously, with no training to do so. Further, the bouts of fetching behavior appeared to be primarily initiated by the cat, rather than the owner [5]. Because this study specifically solicited individuals with cats who fetched, the prevalence of fetching behavior among cats in general could not be inferred.

In another study, cat owners were asked to complete a survey about their cats’ behavior (unrelated to fetching) while in the waiting room of one of four participating veterinary clinics. Participants were invited to list “tricks” that their cat knew, and several noted that their cats fetched. Of the cats represented (n = 819), 135 (16.5%) exhibited fetching behavior [16]. Breed differences were observed, with Persian cats being found to be the least likely to exhibit fetching behavior (9.1% of Persian cats represented) and Abyssinians being most likely to fetch (45.5%; however, the sample size of Abyssinian cats was small, n = 11).

No previous study has examined the prevalence of, or breed-specific differences in, fetching behavior of dogs. A study of Labrador Retrievers registered with the UK Kennel Club, utilized pedigree and a behavior survey (C-BARQ; Canine Behavioral Assessment and Research Questionnaire) to assess the heritability of different traits. There was modest heritability of fetching behavior (*h*^2^ = 0.38) [17]. Another survey-based study of 2020 registered Labrador Retrievers, categorized as either pets, gundogs, or show dogs, used the C-BARQ to measure behavioral traits. There was an effect of working status, with gundogs being more likely to fetch compared to dogs kept as pets or show dogs [2]. It is not known whether this difference was the result of genotypic/phenotypic selection, or training/learning history.

Given the existing gaps in knowledge about fetching behavior in cats and dogs, the goal of the current study was to build on the limited existing research in an effort to better understand the prevalence of the behavior in both species and the respective animal-related factors associated with fetching. We examined the frequency of fetching and toy-carrying behavior and how these might relate to other behaviors in a larger sample of cats. We also compared animals who fetch to those who do not; we explored associations between specific animal-related factors (e.g., sex, age, health, neuter status, etc.) and fetching and toy-carrying behavior. Finally, we assessed whether fetching was associated with other behaviors (e.g., predatory behavior, aggression) in domestic cats and dogs.

For cats, we hypothesized that fetching and toy-carrying behavior would be highly correlated, as cats who fetch would necessarily need to carry items. Because fetching involves multiple aspects of the predatory sequence, we predicted that fetching in cats would be related to play and predatory behaviors.

Because some people may train their pets to fetch, we hypothesized that fetching would be related to trainability in both species. Finally, because fetching may also be a form of social play, we hypothesized that fetching would be correlated with the Fe-BARQ subscale assessing sociability toward humans. A comparable association could not be explored for dogs because the C-BARQ does not contain a human sociability scale.

## Materials and methods

### Ethical review

This study was reviewed by the University of Pennsylvania Institutional Review Board and determined to be exempt from ethical review as it did not meet the criteria for human subjects research. Because no personal information was collected from participating cat owners, formal consent to participate was not required.

### Survey instrument

#### Fe-BARQ

Cat owners could voluntarily complete the Feline Behavioral Assessment and Research Questionnaire (Fe-BARQ; https://vetapps.vet.upenn.edu/febarq/). Data were collected from September 2015 to September 2023.

The Fe-BARQ [18] is a validated, frequently used tool to assess cat behavior. The questionnaire asks owners to describe the frequency of 100 different cat behaviors. The responses are five-point Likert scales ranging from never (0), seldom (1), sometimes (2), usually (3) or always (4). The Fe-BARQ includes three questions relevant to fetching behavior: Item 3 (carries small objects/toys in the mouth to interact with), Item 13 (initiates interactive play with people in the home i.e. brings toys, strings, or small objects to play with), and item 91 (plays ‘fetch’; likes to retrieve thrown objects or toys).

The Fe-BARQ has 23 subscales, including Activity/Playfulness, Sociability with People, and Predatory Behavior. Demographic information about the cat was also collected at the time of the survey including age, sex, neuter status, whether the cat lived indoors only, and whether the cat was declawed. The owner was asked about their experience with cat ownership, and also asked to assess whether their cat had behavior problems (no problems, minor problems, moderate problems, serious problems). The Fe-BARQ subscale scores are calculated by averaging all of the responses to that subscale’s items.

#### C-BARQ

Dog owners could voluntarily complete the Canine Behavioral Assessment and Research Questionnaire (C-BARQ; https://vetapps.vet.upenn.edu/cbarq/). Data were collected from March 2008 to July 2022.

The C-BARQ [19] is a validated, frequently used tool to assess dog behavior. The questionnaire asks owners to describe either the frequency or the severity of 100 different behaviors. The responses are five-point Likert scales ranging from never (0), seldom (1), sometimes (2), usually (3), to always (4) for frequency, and on a spectrum for severity that differed based on the behavior (e.g., absent (0), mild to moderate (1, 2, 3) and severe (4)). The C-BARQ contains one item relevant to fetching behavior, Item 8: “will "fetch" or attempt to fetch sticks, balls, or objects”.

The C-BARQ comprises 14 subscales, including Stranger- and Owner-directed aggression, Excitability, Trainability, and Stranger-directed fear. Demographic information about the dog was also collected at the time of the survey including age, sex, neuter status and where the dog was acquired. The owner was asked about their experience with dog ownership and whether they were concerned about their dog’s health. The C-BARQ subscale scores are calculated by averaging all of the responses to that subscale’s items.

### Data analysis

Data were analyzed in SAS (SAS Institute, Cary, NC). Spearman correlations were run between all survey items, using the 0–4 Likert scale (S1 File). Due to the large dataset, many correlations were statistically significant. We chose to focus on those correlations that were greater than *r* = 0.32, as those would indicate variables where greater than 10% of the variance (*R*^2^ > 0.10) in one variable was explained by the other.

For the simplicity of analysis for regression models and the chi-square test of independence, frequency data for fetching and carrying behaviors was collapsed into two categories. Cats and dogs who never or seldom fetched/carried objects/toys were considered “non-fetchers/non-carriers” and cats and dogs who were described as sometimes, usually, or always fetching or carrying objects/toys were considered “fetchers/carriers”.

Two logistic regressions were conducted with animal-related and environmental factors (sex, if neutered, if declawed, if the cat had behavior or health problems, if the cat was indoors only, the amount of time the cat spent alone, the number of other cats in the household, whether there were dogs in the home, and the cat’s age at the time of the survey) as predictors, and fetch and carry as binary outcomes. For dogs, we used the predictors sex, if neutered, if the dog had health problems, if there were other dogs in the household, and the dog’s age at the time of the survey.

A backwards stepwise regression approach was taken with all models, meaning that all predictors were included in the first model. Any insignificant predictors were removed one at a time, until the remaining model only included items with p-values < 0.05.

To assess the relationship between breed and fetching (25 breeds) and carrying behavior (23 breeds) in cats, and breed and fetching behavior in dogs (230 breeds), separate omnibus chi-square tests were conducted. For the analysis of the Fe-BARQ data, breeds were further retained for the chi-square analysis if each cell contained at least five individuals.

For the analysis of C-BARQ data, fewer than 80% of the cells contained values of five or more [20]. Breeds (n = 230) were retained for chi-square analysis if each cell contained at least five individuals or, if a breed had fewer than five individuals in each cell, then if the total sample for that breed was greater than N = 36. We chose this sample size because a binomial test indicated that based on the overall probability of a dog fetching, with a sample size of 36, having four or fewer dogs who did or did not fetch would be statistically different from chance. Because we included cells with n < 5, we also conducted a Fisher’s exact test with Monte Carlo estimates.

The only breeds that fulfilled this inclusion criteria (a sample of > 36, with an individual cell with < 5) were those where fetching was highly prevalent (Aussiedoodle n = 58 (1 who did not fetch); Belgian Groenendael n = 57 (4 who did not fetch); Bernedoodle n = 37 (3 who did not fetch); Briard n = 45 (3 who did not fetch); Lagotto Romagnolo n = 77 (4 who did not fetch); Mudi n = 39 (4 who did not fetch); and Wirehaired Pointing Griffon n = 38 (4 who did not fetch)).

We identified the cells of interest by calculating an adjusted standardized residual for each [21]. Cells with an absolute value > 2 indicate breeds that show a lack of fit with the null hypotheses, meaning that these breeds were more (>2) or less (< -2) likely than expected to exhibit fetching/carrying behavior.

## Results: Domestic cats

### Fe-BARQ participants

A total of 8224 surveys were assessed. Over half of participants (57.3%) were from the United States. There were surveys from 87 additional countries, with 10.7% of participants from the United Kingdom, 8.5% were from Australia, and 5.3% were from Canada. Every other country represented less than 5% of the total sample.

The most common source of the cat was an animal shelter (n = 2773, 33.7%). Other popular ways by which cats were sourced included being found as a stray (18%) and being obtained from a friend, relative or neighbor (18.4%). Only 8.3% of the cats were purchased from a breeding cattery.

Of the cats represented in the surveys, 3966 were female (48.2%) and 4258 were male (51.8%). Most cats (95.3%) were neutered. Six hundred and forty-one cats were declawed (7.8%). The average age of the cats in the study was 5.5 years (SD: 4.3 years). More than half (53.2%, N = 4378) of the cats were housed indoors exclusively. All others had some form of outdoor access, including 20.3% living indoors but having free access to the outdoors, and 25.1% living indoors and having controlled access to the outdoors.

### Fetching prevalence

Fetching behavior was more common than reported in previous research, with 40.9% of cats being reported to, “sometimes, usually, or always” play fetch. More cats were reported to carry objects than fetch them, with 57.9% of cats being reported to “sometimes, usually, or always” carry toys or small objects. In addition, 39.3% of cats “sometimes, usually, or always” initiated interactive play with people by bringing them toys.

### Logistic regression models for fetching/carrying outcomes

The overall model for fetching behavior was significant, *X*^2^(5) = 432.68, *p* < 0.001. Cats who were kept indoors only and who lived in a home without dogs were more likely to fetch. Those who were female and had health problems were less likely to fetch. The likelihood of fetching decreased with age.

The overall model for carrying behavior was significant, *X*^2^(6) = 743.31, *p* < 0.001. Cats who were neutered, kept exclusively indoors, declawed, and those who lived in a home without dogs were more likely to carry small objects/toys in their mouths. Cats with health problems were less likely to carry objects. The likelihood of object carrying decreased with age.

The overall model for bringing toys was significant, *X*^2^(7) = 550.78, *p* < 0.001. Cats who lived indoors only were more likely to initiate play by bringing toys to their owner. Female cats, cats who had health or behavior problems, and cats who lived with dogs were less likely to initiate play by bringing toys to their owner. The likelihood of initiating play decreased with age and with increased days the cat spent alone.

See Table 1 for Regression results, including beta estimates and odds ratios for each predictor.

**Table 1:** Logistic regression results for factors related to fetching and carrying objects, and initiating play in cats.

Factor	β	*X* ^2^	*p*	OR
Fetching				
Sex: Female	-0.06	7.23	0.007	0.88 [0.80, 0.97]
Health: Has health problems	-0.12	13.13	0.0003	0.79 [0.69, 0.90]
Lives: Indoors only	0.10	18.08	< 0.0001	1.22 [1.14, 1.34]
Lives with: Dogs in home	-0.20	51.02	< 0.0001	0.66 [0.59, 0.74]
Age	-0.002	260.87	< 0.0001	0.998 [0.998, 0.998]
Carrying objects				
Reproductive status: Is Neutered	0.12	4.91	0.027	1.28 [1.03 1.6]
Is Declawed	0.11	6.00	0.014	1.25 [1.05, 1.49]
Health: Has health problems	-0.16	26.08	< 0.0001	0.72 [0.64, 0.82]
Lives: Indoors only	0.12	27.67	< 0.0001	1.28 [1.17, 1.41]
Lives with: Dogs in home	-0.10	11.72	0.001	0.82 [0.74, 0.92]
Age	-0.003	517.03	< 0.0001	0.997 [0.997, 0.998]
Initiates play (brings toys)				
Sex: Female	-0.060	6.49	< 0.0001	0.89 [0.81, 0.97]
Behavior: Has behavior problem	-0.052	4.54	0.033	0.90 [0.82, 0.99]
Health: Has health problem	-0.148	18.75	< 0.0001	0.75 [0.65, 0.85]
Lives: Indoors only	0.161	45.87	< 0.0001	1.38 [1.26, 1.52]
Days alone	-0.041	14.41	0.0001	0.96 [0.94, 0.98]
Lives with: Dogs in home	-0.145	24.32	< 0.0001	0.75 [0.67, 0.84]
Age	-0.002	332.41	< 0.0001	0.998 [0.998, 0.998]

### Correlations between fetching/carrying and other behaviors

Plays Fetch was correlated (*r* > 0.32) with several individual items from the Activity/Playfulness subscale. The highly correlated items were: ACT 03 ‐ Carries small objects/toys in the mouth to interact with (*r* = 0.50), ACT 04 ‐ Runs and jumps in the air (*r* = 0.33), ACT 12 ‐ Initiates mutual chasing by running from room to room in the house (*r* = 0.32), and ACT 13 ‐ Initiates interactive play with people in the home (i.e. brings toys, strings, or small objects to play with (*r =* 0.55).

Plays Fetch was moderately correlated with the Activity/Playfulness subscale (*r* = 0.48). It also had comparatively small correlations with the subscales: Trainability (*r* = 0.23), Predatory Behavior (*r =* 0.17), Prey interest (*r* = 0.23), and Sociability to people (*r* = 0.12).

Carrying behavior and bringing toys to play with had statistically significant but relatively small correlations with the subscales of interest, see Table 2.

**Table 2:** Spearman correlations between plays fetch, carries objects/toys, initiates play and other Fe-BARQ items.

Fe-BARQ Item	Plays Fetch	Carries Objects/ Toys	Initiates Play
Quickly learns how to play with new introduced toys.	**0.33**	**0.40**	**0.35**
Curious: actively investigates/ explores new objects, sights, or changes in its environment.	0.23	**0.34**	0.29
Carries small objects/toys in the mouth to interact with.	**0.50**	-	**0.60**
Runs and jumps in the air.	**0.33**	**0.49**	**0.42**
Engages in active jumping and climbing on high surfaces, furniture or curtains/drapes.	0.24	**0.33**	0.31
Exhibits sudden bursts of running or climbing in certain periods of the day.	0.25	**0.34**	**0.33**
Exhibits sudden jumping and running during playful activity.	0.28	**0.40**	**0.37**
Stalks, chases or pounces on moving objects (string, balls, soft toys, etc.) during playful activity.	0.30	**0.42**	**0.37**
Displays running/chasing and hunting/pouncing on unseen/imaginary prey/objects-	0.25	**0.33**	**0.34**
Chases and ambushes other household members (including pets) playfully.	0.29	**0.39**	**0.39**
Chases or follows shadows or light spots.	0.24	0.31	0.31
Initiates mutual chasing by running from room to room in the house.	0.32	**0.40**	**0.45**
Initiates interactive play with people in the home (i.e. brings toys, strings, or small objects to play with).	**0.55**	**0.60**	-
Plays with other household cat(s)	0.26	**0.35**	**0.36**
Activity Subscale	**0.48**	**0.66**	**0.64**
Trainability Subscale	0.23	0.16	0.23
Predatory Behavior Subscale	0.17	0.24	0.20
Prey Interest Subscale	0.23	0.23	0.23
Sociability with People Subscale	0.12	0.09	0.13

*Note*. All correlations are statistically significant at p < 0.001. Spearman correlations in bold indicate *r* > 0.32.

### Chi-square test for breed-specific effects

For fetching behavior (n = 7733), the omnibus chi-square test was statistically significant, *X*^2^(24) = 89.44, *p* < 0.001. Bengal, Burmese, Siamese, and Tonkinese cats were more likely than expected to fetch. Domestic Longhaired cats were less likely than expected to fetch.

For carrying behavior (n = 7198), the omnibus chi-square test was statistically significant, *X*^2^(22) = 47.23, *p* = 0.001. Abyssinian, Bengal, Siamese, and Siberian cats were more likely than expected to display carrying behavior. Domestic Longhaired and Persian cats were less likely than expected to carry items(see S1 File).

## Results: Domestic dogs

### C-BARQ participants

A total of 73724 surveys were assessed. Over half of participants (53.4%) were from the United States. There were surveys from 150 additional countries, with 6.5% of participants coming from Canada, 5.8% being from the United Kingdom, and 3.1% coming from Australia. Every other country represented less than 3% of the total sample.

The most common source of the dog was from a breeder (n = 31103, 42.2%). Other popular sources of dogs included shelters (30.1%) and friends or relatives (10.6%). Most dogs (59.1%) lived with at least one other dog.

Of the dogs represented in the surveys, 34836 were female (47.2%) and 38888 were male (52.7%). The majority of dogs were neutered (71.6%). Most of the dogs were healthy, with 14% having a health problem. The average age of the dogs in the study was 4.2 years (SD: 3.3 years).

### Fetching prevalence

The overall prevalence of dogs who were reported to “sometimes, usually, or always” play fetch was 77.8%.

### Logistic regression models for fetching outcomes

The logistic regression model for fetching behavior was significant, *X*^2^(5) = 3421.13, *p* < 0.001. Being female, spayed or neutered, having health problems, and living with other dogs all reduced the likelihood of fetching. Fetching behavior decreased with increasing age.

See Table 3 for Regression results, including beta estimates and odds ratios for each predictor.

**Table 3:** Logistic regression results for factors related to fetching in dogs.

Factor	β	*X* ^2^	*p*	OR
Sex: Female	-0.05	25.95	< 0.0001	0.91 [0.88, 0.94]
Reproductive status: Is Neutered	-0.11	91.62	< 0.0001	0.81 [0.77, 0.84]
Health: Has health problems	-0.10	16.55	< 0.0001	0.90 [0.86, 0.95]
Lives with: Dogs in home	-0.14	223.26	< 0.0001	0.75 [0.73, 0.78]
Age	-0.003	2269.20	< 0.0001	0.997 [0.997, 0.998]

### Correlations between fetching and other behaviors

Fetching frequency was not correlated above *r* = 0.32 with any individual C-BARQ items. It was statistically correlated with the Trainability subscale *r* = 0.47, *p* < 0.0001. See Table 4.

**Table 4:** Spearman correlations between plays fetch and other C-BARQ items.

C-BARQ Item or Scale	Plays Fetch
When off the leash, returns immediately when called	0.18
Obeys the "sit" command immediately	0.19
Obeys the "stay" command immediately	0.18
Seems to attend/listen closely to everything you say or do	0.19
Slow to respond to correction or punishment; "thick-skinned"	0.04
Slow to learn new tricks or tasks	0.23
Easily distracted by interesting sights, sounds, or smells	0.05
Will "fetch" or attempt to fetch sticks, balls, or objects	‐‐
Trainability Subscale	**0.47**
Chasing Subscale	0.04
Energy Subscale	0.32
Excitability Subscale	0.10
Attachment/Attention-Seeking Subscale	0.10

*Note*. All correlations are statistically significant at p < 0.001. Spearman correlations in bold indicate *r* > 0.32.

### Chi-square test for breed-specific effects

For fetching behavior by breed (n = 71595), the omnibus chi-square test was statistically significant, *X*^2^(230) = 4625.42, *p* < 0.001, Fisher’s exact test *p* < 0.0001. Forty-five dog breeds were more likely than expected to fetch; 88 dog breeds were less likely than expected to fetch (S1 File). Many of the breeds more likely than expected to fetch included dogs from the Retriever, UK Rural, Poodle, Pointer and Spaniel clades (e.g., Labrador and Golden Retrievers, Border Collies, Australian Shepherd, German Wirehaired Pointer, English Cocker Spaniel). Many of the breeds represented in those less likely to fetch included dogs from the Spitz, Toy, Mediterranean, Terrier, European Mastiff, and UK Rural clades (e.g., Siberian Husky, Chihuahua, Great Pyrenees, Yorkshire Terrier, Rhodesian Ridgeback, Greyhound) [22].

## Discussion

This study provides estimates of the prevalence of fetching behavior in a large sample of owned domestic cats and dogs. The data from these two surveys reveal that fetching behavior may be more common in cats than previously estimated (almost 41% of cats represented in the data) and provides, to our knowledge, the first estimate of fetching behavior prevalence among dogs (almost 78%). The results also support sex, age, and breed differences in fetching behavior in cats and dogs, which we describe in more detail as follows.

### Why fetch?

Given the different domestication history, ethology, and physiology of domestic cats and dogs, these findings raise questions as to why both species fetch, and why the behavior is much more common in dogs than cats.

Predatory behavior involves three phases: search, pursue, and capture [23]. Within these sequences are other behaviors, including orient, eye, stalk, chase, grab-bite, kill-bite, and dissect. Species-specific modifications to the predatory sequence exist and are thought to optimize hunting success for prey type within an ecological niche [24]. In both cats and dogs, fetching behavior overlaps with specific parts of the predatory sequence of behaviors. In dogs, certain behaviors in the predatory motor pattern have been selected for exaggerated or diminished frequency in breeds based on their working role. Coppinger & Coppinger [24] theorized that holding an object in the mouth was comparable to the grab-bite aspect of the carnivore predatory sequence and is hypertrophied (overdeveloped) in some breeds of dogs while kill-bite is underdeveloped or absent. In these breeds, rather than progressing to killing and dissecting, fetching behavior may result [24]. In cats, carrying prey after killing is common [11, 25], and could also be an expected part of the feline predatory sequence.

Thus, both cats and dogs possess the capacity for fetching behavior, which may have been retained and accentuated through domestication, especially in some breeds. We posit four possible explanations for how fetching may have become ingrained in some cats and dogs. These hypotheses are not mutually exclusive nor necessarily exhaustive. However, we believe they present opportunities for further research on fetching behavior in companion animals.

#### Hypothesis: Fetching is a form of object play

In cats, fetching had the highest correlation with the Activity/Playfulness subscale (of which it is an item, *r* = 0.47); in dogs, there was a moderate correlation with the Energy subscale (which may be analogous to the Activity/Playfulness subscale in cats; *r* = 0.32). In cats, object play is believed to represent a rehearsal of innate predatory motor patterns; however, there are still open questions as to whether in captive adult animals (including companion animals), object play is influenced by domestication and human behavior [26] (Hall, 1998).

For cats, the correlations with prey interest (*r* = 0.23) and predatory behavior (*r* = 0.17) were relatively low. In the C-BARQ, the closest measure of predatory behavior is the Chasing subscale, which had a low correlation with fetching (*r* = 0.06). In both species, these findings suggest that fetching is more closely related to playful behavior than it is to predation. This suggestion is also borne out by the observed decline in fetching associated with aging in cats and dogs. The sequence of behavior also fulfills the key criteria for "play" in that it does not appear fully functional and appears to be pleasurable or rewarding [27]. The finding that females were less likely to fetch, and that spay/neuter status was not a significant factor in expression of the behavior further suggests that it may be less associated with predation given that presentation of prey to offspring is typical of maternal behavior and related to social facilitation of offspring hunting/killing behavior.

Cats naturally, without any training, will display discrete behaviors used when hunting prey including “…watching, pouncing, hitting, grasping, pulling down, kicking with hindlegs, tossing and biting” [28]. In the predatory sequence these behaviors occur in an established sequence but in the absence of opportunities to hunt, the behavioral sequence may decouple. Predatory behavior in cats is so highly motivated that even in the absence of hunger it will be expressed, albeit with variable frequency across individuals, and it may occur out of context such as during play with objects [28].

Predatory behavior and prey interest may be influenced by whether the cat has outdoor access and opportunities to hunt. In the current study, being housed exclusively indoors increased the likelihood that a cat would fetch, and in previous studies has increased the likelihood of play behavior [29]. This may be because owners of indoor cats may be more likely to play with them [9]. Alternatively, cats allowed outdoor access may have less motivation to fetch as they have more opportunity to engage in physically active and predatory behavior while outside. If this is the case, fetching in indoor cats might be regarded as a form of environmental auto-enrichment.

While there is little research on dogs’ innate tendencies to fetch, there are several studies that utilize fetching as a cognitive task to measure dogs’ cooperative behaviors [e.g., 30, 31]. The current study identified several breeds that were more likely to fetch, including dogs from the Retriever, UK Rural, Poodle, Pointer and Spaniel clades [22]. Predatory motor patterns in these breeds have hypertrophied chase and/or grab-bite behavior that begins to appear during the critical period of socialization. The association between the onset of this motor pattern and socialization may result in the dog displaying the behavior in the presence of their human companion as a form of social play resulting in fetching [24].

#### Hypothesis: Fetching behavior has been selected for during domestication or breed development

Breed differences in the number and intensity of behaviors in the predatory sequence exhibited have been identified [24], which may explain the breed differences we found in fetching behaviors in dogs. However, almost all breeds of both cats and dogs had some animals who fetched, including wolves and wolf hybrids (S1 File). A comparative study found that mixed-breed and purebred dogs were more likely to chase and retrieve objects than hand-reared wolves [32] and a study of social play and cooperation found that three out of thirteen wolf puppies offered retrieval behavior spontaneously [33]. Together these results suggest a possible influence of domestication, but also that variation in fetching or retrieval-type behavior may have existed prior to domestication.

Differences in breed expression of behaviors within the predatory sequence that are likely due to human selection are outlined in Table 5 [24]. Early in the domestication process, before intensive goal-directed breed development, dogs were selected for specific traits and behaviors that may have aided humans, most notably with herding and hunting [34]. Several types or landraces of working dogs have been identified, including dogs who move livestock, those who guard livestock, sporting dogs that point and/or retrieve killed prey, and hounds that track prey by scent or chase running prey. In the current study, breeds that were identified as high fetchers included herding, pointing, and retrieving breeds while those identified as low fetchers included hound, livestock guarding (LGD), and wildtype breeds.

**Table 5:** Selected behaviors of the predatory sequence in several types of dogs. (Adapted from Coppinger & Coppinger, 2001).

Breed type	Predatory Sequence					
Wild type	Orient>>	eye>>	stalk>>	chase>>	grab-bite>>	kill-bite
Livestock Guardian	(Orient)	(eye)	(stalk)	(chase)	(grab-bite)	(kill-bite)
Header	**Orient**>>	**EYE**>>	**STALK**>>	**CHASE**	(grab-bite)	(kill-bite)
Heeler	Orient>>	eye	stalk	**CHASE**>>	**GRAB-BITE**	(kill-bite)
Hound	Orient>>		**Mark**>>	**CHASE**>>	**GRAB-BITE**>>	**KILL-BITE**
Pointer	**Orient**>>	**EYE**	(stalk)	(chase)	**GRAB-BITE**	(kill-bite)
Retriever	**Orient>>**	eye	stalk	chase	**GRAB-BITE**	(kill-bite)

*Note*. >> = connected motor patterns **bold** = hypertrophied () = fault/absent

Breeds developed to move livestock including headers (e.g., Border Collie), and heelers (e.g., Australian Shepherd, Australian Cattle Dog) and breeds developed as hunting companions including those in the Pointer group (e.g., German Shorthaired Pointer, Vizsla, Weimaraner) and Retriever group (e.g., Labrador Retriever, Golden Retriever), were more likely to fetch. Conversely, breeds in the Hound group including both scent hounds (e.g., Dachshund, Black and Tan Coonhound, Blue Tick Coonhound, Beagle) and sighthounds (e.g., Afghan Hound, Irish Wolfhound, Greyhound) were found to be low fetchers.

A possible explanation for these differences is that dogs identified as high fetchers may get ‘stuck’ in a repetitive pattern of chase >> grab-bite, and fetch, because the predatory motor pattern is truncated [24]. In comparison, low fetching breeds such as hounds have also been selected for hypertrophied chase, grab-bite, and kill-bite and maintain the link from grab-bite to kill-bite. Thus, they can complete the behavior sequence and do not get ‘stuck.’ In the case of LGD, they lack the predatory motor pattern entirely and fetching would be expected to be rare.

Because they are the most common, non-purebred cats (e.g., domestic shorthair, domestic longhair) represented the majority of fetching cats. However, they are not more likely than expected to fetch, and in agreement with Forman et al. [5] and Voith and Borchelt [16], we found breed differences in the prevalence of fetching behavior in cats. The three purebred breeds more likely than expected to fetch (Siamese, Burmese, and their crossbreed, the Tonkinese) are part of a shared origin in the Far East [35], a cluster of cats that are genetically different from cats from other regions of the world (e.g., the Mediterranean Basin, Western Europe, Africa) [36]. Geneticists believe that domestic cats were brought to the Far East early in the process of cat domestication, and then were genetically isolated from other populations for an extended period [36]. The greater prevalence of fetching in this population could be due to a founder effect, genetic drift, or be the result of increased local selection pressures for the tendency to engage in fetching behavior in this region of the world.

Bengals, a hybrid recently developed by crossing the Asian Leopard cat with the domestic cat, were also more likely to fetch than most other breeds. They are described as a very active breed that displays high levels of hunting behavior [37]. While cats are often considered to be ‘self-domesticated’ [38], the breed differences in fetching raise questions about possible working roles for cats in former times. Cats were commonly depicted as accompanying their owners on bird hunts in Egyptian tomb paintings from circa 1450 BCE [39], raising the intriguing possibility that they may have been used by humans for retrieving or chasing small prey items during their early history.

#### Hypothesis: Fetching is a type of social play

The domestication process has resulted in the retention of juvenile behaviors into adulthood, specifically increasing prosocial behaviors such as playfulness and sociability and decreased fearfulness and aggressiveness [40]. Companion animals also use body language to indicate their desire for social interactions. For example, dogs offer a play bow to indicate intention to play with other dogs or humans [41, 42]. Kittens have documented play solicitation signals with each other, such as a “side step” and the “open mouth” play face [43].

Early life experiences during each species’ sensitive period may also have an impact on tendency to play with humans, and what forms that play may take. For example, when observed during play with a human caretaker from 6 weeks to approximately one year of age, puppies in a guide dog program became gentler and more self-controlled over time with their human caretaker, suggesting that the puppies learned to cooperate [44]. In kittens, play behavior toward a moving string toy was reduced by the kittens’ fear of humans [45].

In both species, initiating fetching by bringing a toy to a human caretaker appears to be an invitation to play. It is assumed that play with humans is a social activity for dogs [41] and this has recently been explored in cats [9]. Forman et al. [5] reported that fetch behavior was often initiated spontaneously by cats, and fetching was more frequent and resulted in more object retrievals when cats initiated it.

If fetching is a form of social behavior, it would be expected to correlate with measures such as attention-seeking or sociality. However, this was not the case for either cats or dogs in the current study. Some possible explanations include that sociality may only explain a small component of fetching behavior, or that the specific questions in the surveys examined did not permit assessment of the social aspects of fetching behavior. Alternatively, the animal may simply be using the human to perpetuate an inherently rewarding game of chase and catch. The fact that the human also finds the game rewarding may be irrelevant from the animal’s perspective. The possibility that the level of expression of fetching in cats may relate to the strength of the human-animal bond should be explored. While the surveys examined did not permit examination of such an association, understanding of whether such a relationship exists would give greater insight into the potential motivation for fetching behavior in cats.

#### Hypothesis: Fetching has been reinforced by training or learning effects

We anticipated a stronger correlation between fetching and trainability in dogs, because dog owners are more likely to provide training than cat owners and often use play as a reinforcer [e.g., 46, 47]. In the C-BARQ survey, fetching was part of the Trainability subscale derived from Exploratory Factor Analyses and had a moderate correlation with the overall Trainability score. Fetching was not highly correlated with any of the individual items on the subscale (0.04 < *r* < 0.23). For cats, the Trainability subscale has three items, and fetching had similar correlations with the individual items (“Comes when called” (*r* = 0.18), “Readily responds to simple commands” (*r* = 0.19), and “Attends and listens closely” (*r* = 0.23). Thus, it appears that the relationship between trainability and fetching behavior may be similar (and tenuous) in both species. Our findings further support those of Forman et al. [5] that fetching in cats is not likely due to training.

A recent study of behavioral genetics utilizing data from 18385 dogs enrolled in the Darwin’s Dog project reported on two behaviors of interest to the current study, toy-directed motor-patterns (e.g. grab-bite, chase) and biddability (how readily the dog responds to human direction, especially in the training context) [48]. Breeds found to have the highest levels of toy-directed motor behavior were German Shepherd Dogs, Golden Retrievers, and Labrador Retrievers. Breeds with the lowest levels of toy-directed behavior included Great Pyrenees, Malamute, and Greyhound. The breeds identified are similar to those identified as high and low fetchers; our data indicated that German Shepherds, Labrador Retrievers and Golden Retrievers were among the breeds more likely than expected to fetch and had the highest standardized residuals (>13.82; S1 File). Great Pyrenees, Malamutes, and Greyhounds all had standardized residuals of less than -2, indicating that they were less likely than expected to fetch.

Further, in the Darwin’s Dog study, the behavioral trait with the highest heritability was retrieving (52.5 +/- 9.2%) and the factor with the highest heritability was human sociability (67.3 +/- 12%). Breeds found to have the highest levels of biddability were German Shepherd Dogs, Golden Retrievers, and Labrador Retrievers. Breeds with the lowest levels of biddability were Great Pyrenees, Malamute, and Basset Hound (less likely than expected to fetch, see S1 File). Herding breeds were reported to be more toy-directed and biddable while sporting breeds were more toy-directed. These results suggest that, in dogs, biddability and training may have an effect on frequency of fetching especially in breeds predisposed to exhibit the behavior. It is likely that fetching behavior, similar to other behavioral characteristics, is complex, influenced by several interacting environmental and genetic factors in both species [e.g., 48].

Fetching behavior can certainly be reinforced and shaped by human responses. For example, if a cat or dog presents a toy to a human, and the human responds by tossing the toy and engaging in play with their pet, this is likely to be rewarding to the animal. If the owner does not recognize that the animal is soliciting play, fetching behavior may be ‘extinguished’ due to lack of reinforcement. The human movement of tossing a toy may serve as an indicator (discriminative stimulus) of the human’s intention to participate in the fetching game [49] and chasing the thrown object could trigger a predatory sequence that is likely rewarding [50]. The cat or dog is then further reinforced (via praise, petting, attention, or the continuation of the game) when they retrieve the item [49]. However, we are still left with the question of how the fetching behavior began in the first place.

#### Factors that decreased fetching

Our analyses also identified factors that were associated with decreased likelihood of fetching behavior. Older cats and dogs were less likely to be reported as fetching, suggesting a possible association with general play and activity, which also tend to decrease with age [18, 51]. Animals with health issues were also less likely to fetch. Health issues could have a similar effect as aging, and it is also possible that owners interact with their older or unwell cats and dogs differently, generally engaging in less play and activity.

Female animals of both species were also less likely to fetch. This may seem surprising, given the tendency of female cats to carry weakened prey to their kittens to aid them in learning to hunt [11]. All predators must hunt to survive, and sex has not been found to be a significant factor in hunting or related behaviors in cats (e.g., Ceccheti, personal communication, 2021) [52, 53] or dogs [e.g., 54, 55]. However, sex differences in activity or playfulness could also explain this finding.

In dogs, there was an association between being neutered and being less likely to fetch. Neutered cats were less likely to carry objects, but there was no relationship with fetching. However, many more cats in our study were neutered than dogs (95.3% vs 71.5%). It is possible that a larger sample size of unneutered cats would have resulted in a different relationship between desexing and fetching behavior.

For both cats and dogs, living with dogs reduced fetching behavior. This could be due to changes in human-animal interactions in cat-dog households, or cats may be more comfortable in homes without dogs [56]. Cats in households with dogs may be inhibited from developing or expressing fetching behavior due to the behavior of the dog (e.g., if the dog chases or expresses predatory behavior toward cats or objects the cat may want to play with).

The effects of living in a multi-dog household on dog behavior may be highly dependent on the relationship between the dogs; if the relationship is tense, play behavior may be negatively impacted [57] but if the relationship is positive dogs may engage in more conspecific play in multi-dog households. In either case, it is also possible that people are less likely to observe individual behaviors in their pets when there are dogs in the home.

We also posit that brachycephaly may inhibit fetching behavior, as indicated by our breed analyses. Brachycephalic dogs (Pugs, Shih Tzu, Bulldog, Cavalier King Charles Spaniel, Mastiff, Brussels Griffon, Japanese Chin, Lhasa Apso, Pekinese, Chow Chow) and cats (Persians) were less likely to fetch or carry objects (the exception was Boston Terriers). If certain physical traits (e.g., bite and occlusal pattern) aid or hinder predation, it is possible that selection for extreme brachycephaly restricts the ability to perform certain parts of the predatory sequence [e.g., 24, 58] or constrains play behavior in other ways.

### Limitations

This study used data from a self-selected sample of cat and dog owners who were willing to fill out a survey and may not be representative of the overall pet population. However, the invitation for people to participate did not specify that there would be questions about specific behaviors, making it less likely that there would be a strong bias toward over- or under-reporting of fetching behavior in the data.

Unfortunately, the two surveys we utilized in this research were not directly comparable. limiting our ability to conduct identical analyses for both species. Further studies should look at dog behaviors that are not included in the C-BARQ survey that were correlated with fetching behaviors in cats. For example, fetching in cats was correlated with tendencies to both carry objects/toys and initiate play by bringing toys to humans. Demographic questions could be matched to better assess the impact of the environment and owner-related factors. Finally, future research could explore the impact of the owner’s behavior on a pet’s tendencies to fetch.

Some of the effect sizes in our regression analyses were small, especially age and sex. Although we limited analyses to correlations greater than *r* = 0.32, most correlations between variables would be considered low. There are likely many other factors related to fetching behavior that were not measured in this study.

Fetching appears to be largely spontaneous in the domestic cat. Forman et al. [5] found that only around 6% of cats who fetch were trained to do so. The extent to which dogs are trained to fetch is also unknown but is likely much higher. This study did not distinguish trained from spontaneous fetchers in either species. Future studies could explore the effects of human training on fetching behavior.

## Conclusions

Fetching is observed in both cats and dogs, and at higher rates in cats than previously reported (40.9%). Some fetching was observed in most breeds of cats and dogs, although some breeds were more likely to fetch than others. Fetching was more prominent in cat breeds originating in the Far East and in dog breeds from the Retriever, UK Rural, Poodle, Pointer and Spaniel clades. We have proposed multiple hypotheses and open questions for future research as to why fetching behavior may be observed in both companion species, despite their different domestication histories.

## Supporting information

S1 File Supplementary data. Standardized residuals, frequencies, and correlation tables for Fe-BARQ and C-BARQ data.
(XLSX)
